# Supplementary material for: Nuclear retention of unspliced HIV-1 RNA as a reversible post-transcriptional block in latency
Source: Nat Commun. 2025 Feb 28;16:2078. doi: 10.1038/s41467-025-57290-y (PMC11871326; doi:10.1038/s41467-025-57290-y)
Supplement: Supplementary file 1 — Supplementary Information [file 41467_2025_57290_MOESM1_ESM.pdf]

Title: Nuclear retention of unspliced HIV-1 RNA as a reversible post-transcriptional block in latency

Agnieszka Dorman[1,2#], Maryam Bendoumou [3#], Aurelija Valaitiene[4], Jakub Wadas[1,2], Haider Ali[1,2], Antoine Dutilleul[3], Paolo Maiuri[5], Lorena Nestola[3], Monika Bociaga-Jasik[6], Gilbert Mchantaf[7,8,9], Coca Necsoi[10], Stéphane De Wit[10], Véronique Avettand-Fenoël[7,8,9], Alessandro Marcello[11], Krzysztof Pyrc[12*], Alexander O. Pasternak[4*§], Carine Van Lint[3*§] & Anna Kula-Pacurar[1*§]

**Supplementary Information**

**Including:**

**Supplementary Figures 1, 2, 3, 4 and 5 with corresponding Figure Legends.**

**Supplementary Table 1.**

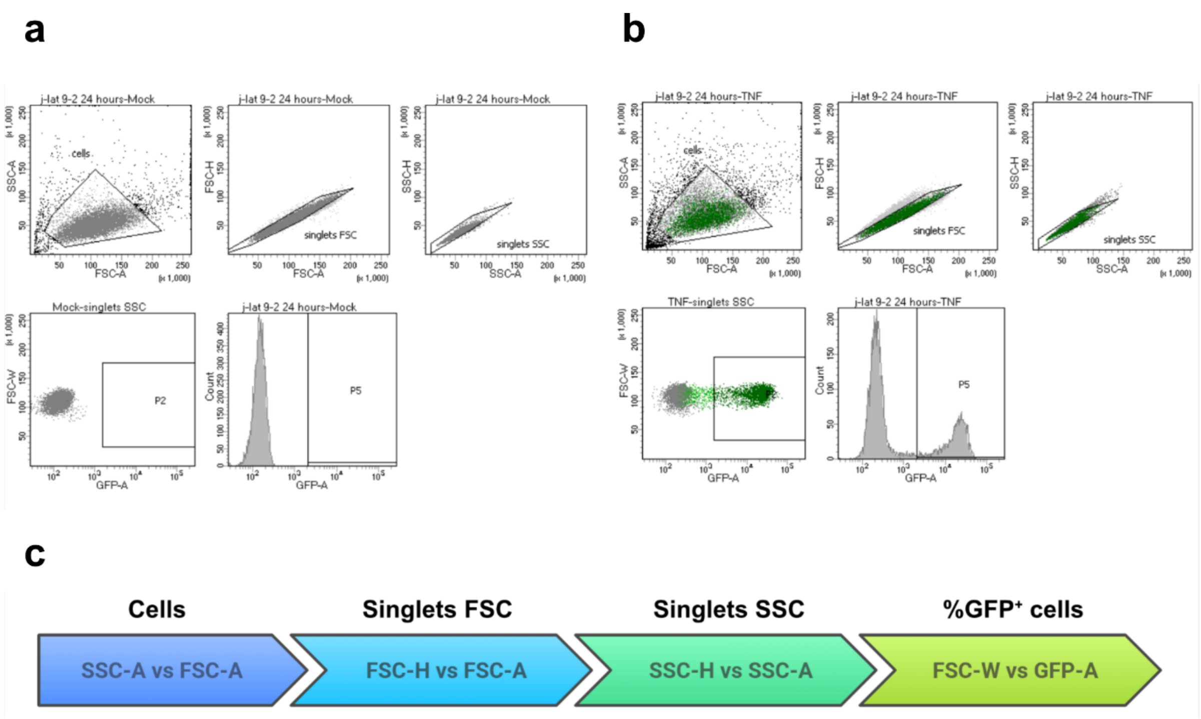


**Supplementary Fig. 1.** Gating strategy to quantify the percentages of GFP-positive J-Lat 9.2 cells. J-Lat 9.2 cells were left untreated as mock sample (**a**) or treated with TNFα [10 ng/ml] (**b**) and after 24 h subjected to flow cytometry gating strategy (**c**) to quantify percentages of GFP$^{+}$ cells.

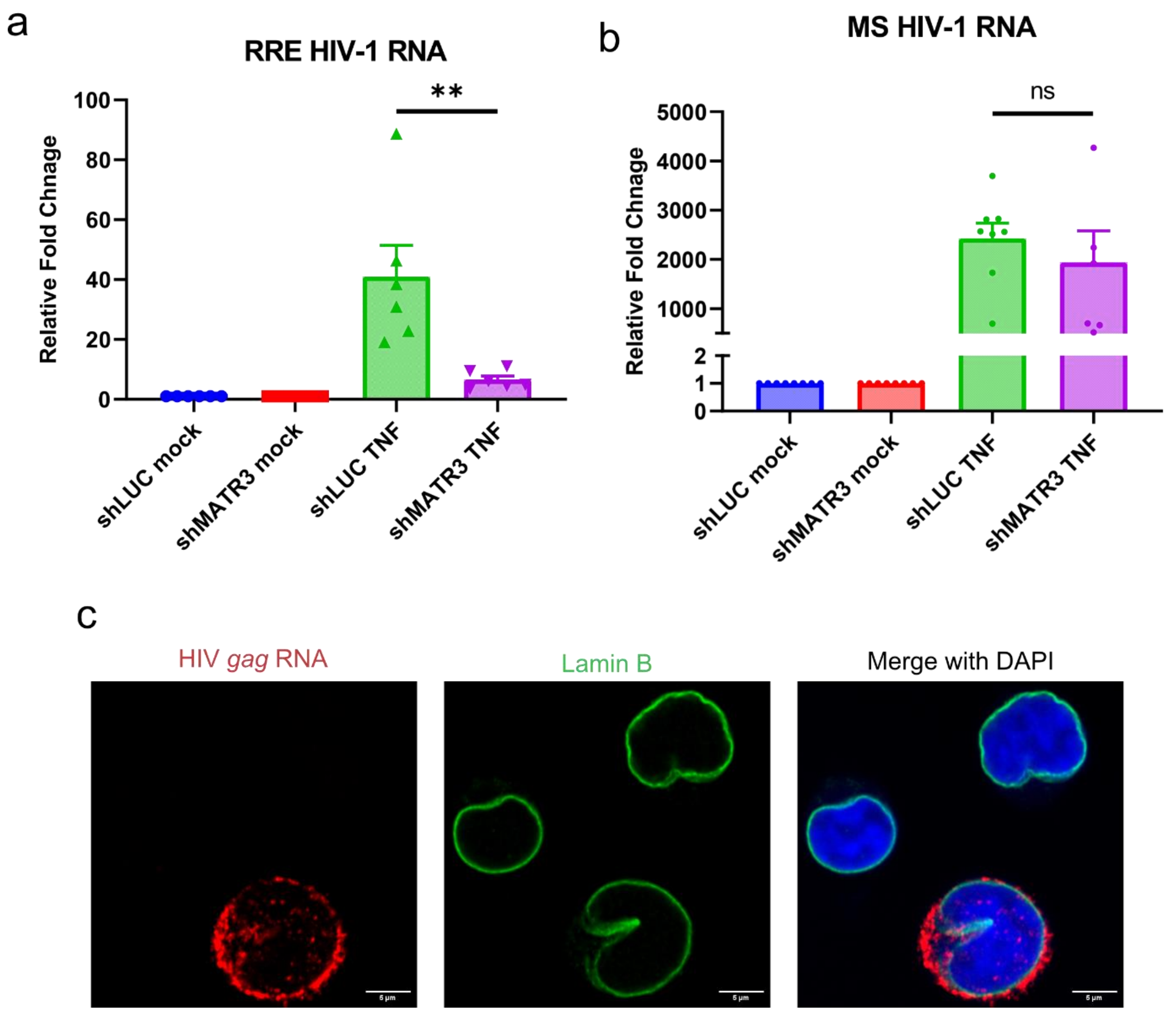


**Supplementary Fig. 2. (a-b)** MATR3 depletion in J-Lat 9.2 cells was obtained with shMATR3 or shLUC lentiviral transduction and three days after puromycin selection, cells were treated with TNFα [10 ng/ml] and after 24 h cells were subjected to intracellular total RNA isolation following RT-qPCR measurements of RRE-containing **(a)** and MS **(b)** HIV-1 transcripts. Values were normalized using 18S RNA primers and were presented as fold changes relative to the values measured in the untreated (mock) shLUC sample, which was arbitrarily set at a value of 1. Results are shown as mean values ± SEM, n=3 (**a**) and n=4 (**b**) in duplicates. Statistics are performed using two-tailed paired t-test, statistical comparisons are indicated if p<0.05, 'ns', not significant. **(c)** J-Lat 9.2 cells were treated with TNFα [10 ng/ml] for 24 h and subjected to Immuno-RNA FISH targeting HIV-1 $^{gag}$RNA, immunostaining using antibodies against Lamin β1, and nuclei staining with DAPI followed by confocal microscopy analysis. HIV-1 $^{gag}$RNA is shown in red, lamin β1 in green, and both channels were merged together with DAPI-stained nucleus shown in blue.

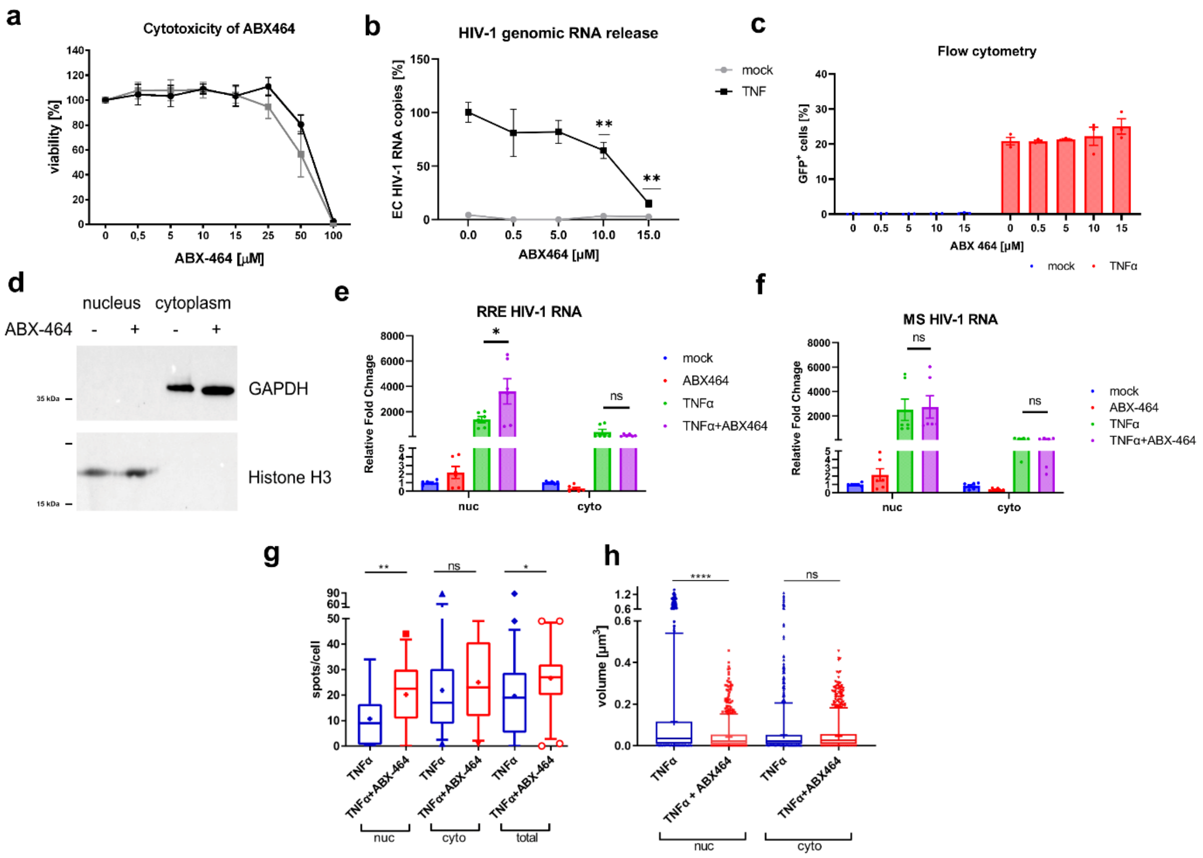


**Supplementary Fig. 3. (a-c)** J-Lat 9.2 cells were treated with TNFα [10 ng/ml] and after 6 h with increasing doses of ABX-464 as indicated. At 18-hours post-treatment cells were subjected to **(a)** cytotoxicity assay (XTT), **(b)** RNA isolation from the supernatant followed by RT-qPCR for genomic HIV-1 RNA (primers targeting HIV-1 gag) or **(c)** flow cytometry analysis to quantify the percentages of GFP+ cells. **(d-f)** J-Lat 9.2 cells were treated with TNFα [10 ng/ml] and after 6 h with ABX-464 [15 µM]. 18 h after ABX-464 stimulation cells were subjected to biochemical nuclear-cytoplasmic fractionation protocol. Nuclear and cytoplasmic fractions were subjected to **(d)** immunoblotting using anti-histone H3 and anti-GAPDH antibodies, or **(e-f)** RNA isolation followed by quantitative real-time RT-qPCR targeting RRE-containing **(e)** and MS **(f)** HIV-1 transcripts. Values were normalized using 18S RNA primers and were presented as fold changes to the values measured in the untreated nuclear and cytoplasmic samples which were arbitrarily set at a value of 1. All results are shown as mean values ± SEM from n=3 in duplicates. Statistics are performed using two-sample t-test. **(g-h)** TNFα and ABX-464-treated J-Lat 9.2 cells were subjected to immuno-RNA FISH targeting HIV-1 gag RNA followed by confocal microscopy analysis. The number **(g)** and volume **(h)** of nuclear, cytoplasmic, and total HIV-1 gag RNA were quantified inside and outside of the nucleus in z-stacks obtained from approx. 10 images/experiment, n=3. Results are presented as box and whiskers with 5-95% confidence interval. Median value is shown as a bar, dots are points outside whiskers representing outliers, mean value is shown as "+". Statistics were performed using the two-tailed unpaired t test. Statistical comparisons are indicated if p≤0.5 (*), p≤0.01 (**), p≤0.001 (***) and 'ns' indicates not significant.

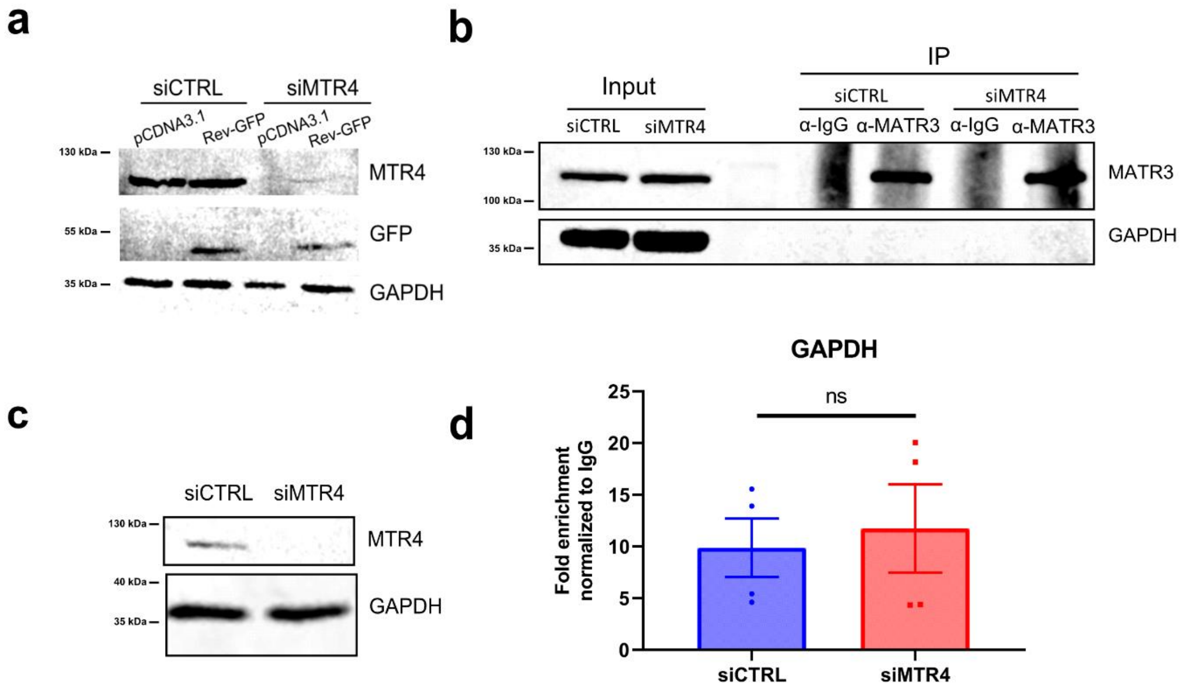


**Supplementary Fig. 4.** (**a**) MTR4 depletion in 293T cells was obtained using siRNA transfection. After 24 h, cells were transfected with pRev-GFP or pCDNA3.1 empty plasmid and after 24 h collected for immunoblotting using anti-MTR4, anti-GFP and anti-GAPDH antibody as a control. **(b-d)** MTR4 depletion in 293T cells was obtained using siRNA transfection. After 24 h, cells were transfected with plasmids pHIV-INTRO, pTat, and pRev-GFP and after 24 h subjected to RNA immunoprecipitation protocol using anti-MATR3 and anti-IgG isotype antibodies. **(b)** Input samples were subjected to immunoblotting with the use of anti-MTR4 and anti-GAPDH antibodies as a control. **(c)** Input and IP samples were subjected to immunoblotting using anti-MATR3 and anti-GAPDH antibodies as a control. **(d)** RNA was purified form IPs and subjected to RT-qPCR targeting *gapdh* mRNA. Values were normalized to input and shown as fold enrichment to IgG control. Results are shown as mean values ± SEM, n = 2 in duplicates. Statistics are performed using two-tailed paired *t* test, ‘ns’ indicates not significant.

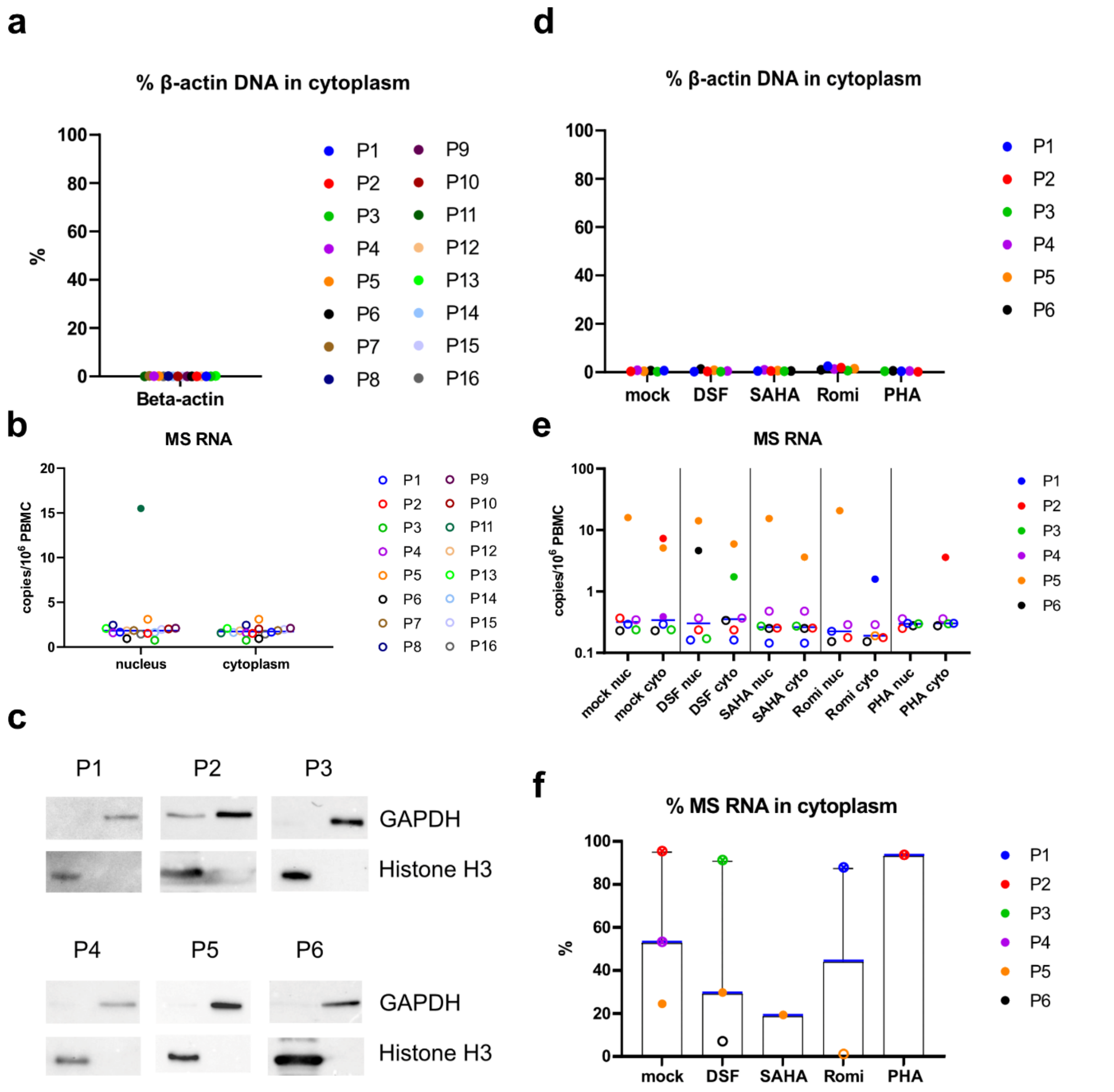


**Supplementary Fig. 5. (a-b)** Freshly isolated PBMCs from 16 ART-treated PWH were subjected to nucleocytoplasmic fractionation for subsequent nucleic acid isolation and qPCR quantification of β-actin DNA (**a**) shown as percentages of β-actin DNA in the cytoplasmic fraction and MS HIV-1 RNA (**b**) shown as copies/$10^6$ PBMCs. **(c-f)** CD8+-depleted PBMCs from 6 ART-treated PWH were mock treated, treated with SAHA [0.5 µM], disulfiram [0.5 µM], romidepsin [17.5 nM] or PHA [1.66 µg/ml] as a positive control in the presence of ARV [280 nM ritonavir, 180 nM azidothymidine, 200 nM raltegravir, 100 nM efavirenz]. **(c)** Three days post-treatment, mock-treated samples were subjected to nuclear and cytoplasmic fractionation and the purity of fractions was analysed by immunoblotting with anti-GAPDH and anti- histone H3 antibodies. **(d-f)** Total nucleic acids were isolated from nuclear and cytoplasmic fractions. β-actin DNA **(d)** and MS HIV-1 RNA **(e, f)** levels were measured by qPCR or RT-qPCR, respectively. Percentages of β-actin DNA in the cytoplasmic fraction are shown in panel **(d)**. Levels of MS RNA in the fractions are shown in panel (**e**) and percentages of MS RNA in the cytoplasmic fraction are shown in panel

**(f)**. (**b, e, f)** Medians are indicated as horizontal lines, and for panel **(f)** interquartile ranges are indicated as well. MS RNA copy numbers were normalized to the cell numbers measured by the beta-actin qPCR assay. Open symbols in panel **(b, e)** indicate undetectable measurements of MS RNA that were assigned the values corresponding to 50% of the corresponding assay detection limits. The detection limits depended on the amounts of the normalizer (input cellular DNA) and therefore differed between samples. Open symbols in panel **(f)** indicate samples where MS RNA in the cytoplasmic fraction was undetectable and censored to 50% of the detection limits; because MS RNA in the nuclear fraction in these samples was detectable, these circles depict the upper limits of the percentages of US RNA in the cytoplasm. Open crossed circles in panel **(f)** indicate samples where MS RNA was undetectable in the nuclear fraction and censored to 50% of the detection limits; because MS RNA in the cytoplasmic fraction in these samples was detectable, these circles depict the lower limits of the percentages of MS RNA in the cytoplasm. Percentages of MS RNA in the cytoplasmic fraction are not shown if MS RNA was undetectable in both nuclear and cytoplasmic fractions.

| **Patient** | **Year of birth** | **Year of 1st treatment** | **Year of blood sampling** | **$CD4^+$ nadir (cells/$mm^3$)** | **$CD4^+$ T cell count (cell/$mm^3$)** | **Viral load** | **Last treatment** |
|---|---|---|---|---|---|---|---|
| P1 | 1996 | 2017 | 2021 | 1051 | 1487 | <20 | TAF/FTC/EVG/COBI |
| P2 | 1985 | 2017 | 2021 | 832 | 960 | 0 | DTG/3TC |
| P3 | 1987 | 2021 | 2021 | 766 | 1288 | 0 | TAF/FTC/BIC |
| P4 | 1974 | 2014 | 2021 | 190 | 290 | 0 | TDF/FTC/DTG |
| P5 | 1985 | 2016 | 2021 | 157 | 605 | 0 | TAF/FTC/EVG/COBI |
| P6 | 1981 | 2020 | 2021 | 502 | 604 | <20 | DTG/3TC |
| P7 | 1970 | 2014 | 2021 | 328 | 813 | <20 | TAF/FTC/RPV |
| P8 | 1985 | 2016 | 2021 | 591 | 880 | 0 | TAF.FTC/RPV |
| P9 | 1988 | 2018 | 2021 | 580 | 580 | 0 | TAF/FTC/EVG/COBI |
| P10 | 1955 | 2011 | 2021 | 231 | 587 | 0 | DTG/RPV |
| P11 | 1957 | 2008 | 2021 | 8 | 973 | 0 | TAF/FTC/EVG/COBI |
| P12 | 1990 | 2011 | 2021 | 342 | 656 | 0 | TAF/FTC/DRV/Cobi |
| P13 | 1979 | 2020 | 2021 | 429 | 831 | 0 | TAF/FTC/RPV |
| P14 | 1985 | 2015 | 2021 | 579 | 740 | 0 | TAF/FTC/DTG |
| P15 | 1988 | 2014 | 2021 | 320 | 936 | <20 | TAF/FTC/DTG |
| P16 | 1973 | 2009 | 2021 | 293 | 662 | 0 | TAF/FTC/RPV |
| P17 | 1992 | 2019 | 2021 | 880 | 1240 | 0 | TAF/FTC/RPV |
| P18 | 1976 | 2015 | 2021 | 305 | 546 | 0 | TAF/FTC/RPV |

| | | | | | | | |
|---|---|---|---|---|---|---|---|
| P19 | 1988 | 2016 | 2021 | 340 | 1280 | 0 | ABC/3TC/DTG |
| P20 | 1983 | 2019 | 2021 | 520 | 790 | 0 | TAF/FTC/EVGc |
| P21 | 1975 | 2020 | 2021 | 25 | 240 | 0 | TAF/FTC/BIC during 6 m, TDF/FTC/DTG |
| P22 | 1984 | 2015 | 2021 | 560 | 1080 | 0 | TAF/FTC/DTG |

**Supplementary Table 1**. Characteristics of patients from the Jagiellonian University Medical College (Krakow, Poland).
