# Supplementary material for: Nuclear retention of unspliced HIV-1 RNA as a reversible post-transcriptional block in latency
Source: Nat Commun. 2025 Feb 28;16:2078. doi: 10.1038/s41467-025-57290-y (PMC11871326; doi:10.1038/s41467-025-57290-y)
Supplement: Supplementary file 2 — Description Of Additional Supplementary File [file 41467_2025_57290_MOESM2_ESM.pdf]

**Description of Additional supplementary file**

**Supplementary data 1:**

Oligonucleotides sequences used in this study.
