# Supplementary material for: Nuclear retention of unspliced HIV-1 RNA as a reversible post-transcriptional block in latency
Source: Nat Commun. 2025 Feb 28;16:2078. doi: 10.1038/s41467-025-57290-y (PMC11871326; doi:10.1038/s41467-025-57290-y)
Supplement: Supplementary file 4 — Reporting summary [file 41467_2025_57290_MOESM4_ESM.pdf]

nature portfolio

Corresponding author(s): Anna Kula-Pacurar

Last updated by author(s): Feb 14, 2025

Reporting Summary

Nature Portfolio wishes to improve the reproducibility of the work that we publish. This form provides structure for consistency and transparency in reporting. For further information on Nature Portfolio policies, see our Editorial Policies and the Editorial Policy Checklist.

Statistics

For all statistical analyses, confirm that the following items are present in the figure legend, table legend, main text, or Methods section.

n/a	Confirmed	
☐	☒	The exact sample size (n) for each experimental group/condition, given as a discrete number and unit of measurement
☐	☒	A statement on whether measurements were taken from distinct samples or whether the same sample was measured repeatedly
☐	☒	The statistical test(s) used AND whether they are one- or two-sided<br>*Only common tests should be described solely by name; describe more complex techniques in the Methods section.*
☒	☐	A description of all covariates tested
☒	☐	A description of any assumptions or corrections, such as tests of normality and adjustment for multiple comparisons
☐	☒	A full description of the statistical parameters including central tendency (e.g. means) or other basic estimates (e.g. regression coefficient) AND variation (e.g. standard deviation) or associated estimates of uncertainty (e.g. confidence intervals)
☐	☒	For null hypothesis testing, the test statistic (e.g. F, t, r) with confidence intervals, effect sizes, degrees of freedom and P value noted<br>*Give P values as exact values whenever suitable.*
☒	☐	For Bayesian analysis, information on the choice of priors and Markov chain Monte Carlo settings
☒	☐	For hierarchical and complex designs, identification of the appropriate level for tests and full reporting of outcomes
☒	☐	Estimates of effect sizes (e.g. Cohen's d, Pearson's r), indicating how they were calculated

Our web collection on statistics for biologists contains articles on many of the points above.

Software and code

Policy information about availability of computer code

Data collection	All software used to collect data is commercially available. Western blot images were captured with an Azure Biosystems C300 imager. qPCR measurements were taken by a Bio-rad C1000 real time. Microscopy images were aquired by confocal microscope ZEISS LSM 880 with 100x/1.46 NA Plan Apochromat Objective with oil immersion and the ZEN Imaging Software (ZEISS). Flow cytometry measurments were performed on BD FACS Fortessa.
Data analysis	All analyses were performed using commercially available software, detailed in the Materials and Methods section. The manuscript was composed in Microsoft Office and figures designed in Affinity Designer 2 or GraphPad Prism 9. Statistical tests and data visualization was performed using GraphPad Prism.

For manuscripts utilizing custom algorithms or software that are central to the research but not yet described in published literature, software must be made available to editors and reviewers. We strongly encourage code deposition in a community repository (e.g. GitHub). See the Nature Portfolio guidelines for submitting code & software for further information.

Data

Policy information about availability of data

All manuscripts must include a data availability statement. This statement should provide the following information, where applicable:

- Accession codes, unique identifiers, or web links for publicly available datasets
- A description of any restrictions on data availability
- For clinical datasets or third party data, please ensure that the statement adheres to our policy

Provide your data availability statement here.

Research involving human participants, their data, or biological material

Policy information about studies with human participants or human data. See also policy information about sex, gender (identity/presentation), and sexual orientation and race, ethnicity and racism.

Reporting on sex and gender	We reported the year of birth of the participants (Suppl. Table 1). Gender is not reported as we did not have access to this information.
Reporting on race, ethnicity, or other socially relevant groupings	n/a
Population characteristics	n/a
Recruitment	We selected 22 HIV-1-infected individuals at the Jagiellonian University Medical College (Krakow, Poland) based on the following criteria: all participants were treated with cART for at least 6 months, had an undetectable plasma HIV-1 RNA level (20 copies/ml) for at least 6 months and had a level of CD4+ T lymphocytes higher than 200 cells/mm3 of blood. Characteristics (year of birth, duration of therapy, nadir CD4+ T cell count, CD4+ nadir, antiviral regimens) of PWH from the Jagiellonian University Medical College are presented in the Supplementary Table 1.
Ethics oversight	Ethical approval (Approval No. 1072.6120.184.2018) was granted by the Human Subject Ethics Committees of the Jagiellonian University Medical College (Krakow, Poland).

Note that full information on the approval of the study protocol must also be provided in the manuscript.

Field-specific reporting

Please select the one below that is the best fit for your research. If you are not sure, read the appropriate sections before making your selection.

☒ Life sciences ☐ Behavioural & social sciences ☐ Ecological, evolutionary & environmental sciences

For a reference copy of the document with all sections, see nature.com/documents/nr-reporting-summary-flat.pdf

Life sciences study design

All studies must disclose on these points even when the disclosure is negative.

Sample size	Experiments done with cell line J-Lat 9.2 were performed at least three times in duplicates, or as indicated in the figure legends. For studies using primary CD4+ T cells obtained from human donors, sample size was at least from ≥3 biological repetitions in duplicates, or as indicated in the figure legends. The sample size was determined based on the minimum requirement to perform statistical tests and the availability of materials.
Data exclusions	For Fig. 2f: cut-off for >1000 spots and >10 micrometer volume.
Replication	Replication details for each experiment are provided in the figure legends. Experiments were typically conducted in triplicate biological repetitions or more in duplicates.
Randomization	Samples were independently and randomly analyzed.
Blinding	Samples were blindly analyzed.

Reporting for specific materials, systems and methods

We require information from authors about some types of materials, experimental systems and methods used in many studies. Here, indicate whether each material, system or method listed is relevant to your study. If you are not sure if a list item applies to your research, read the appropriate section before selecting a response.

Materials & experimental systems

n/a	Involved in the study
☐	☒ Antibodies
☐	☒ Eukaryotic cell lines
☒	☐ Palaeontology and archaeology
☒	☐ Animals and other organisms
☐	☒ Clinical data
☒	☐ Dual use research of concern
☒	☐ Plants

Methods

n/a	Involved in the study
☒	☐ ChIP-seq
☐	☒ Flow cytometry
☒	☐ MRI-based neuroimaging

Antibodies

Antibodies used	Antibodies for western blotting: against MATR3 were purchased from Bethyl (1:2000, #A300-591A, rabbit) or Sigma (1:1000, MABN1587, mouse); MTR4 (1:500, #A300-614A) were purchased from Bethyl; PSF (1:4000; #P2860) and FLAG (1:1000; #F1804) were purchased from Sigma; EXOSC10 (1:1000; #ab50558) were purchased from Abcam; GFP (1:1000, #2555), GAPDH (1:4000, #2118) and H3 histone (1:5000; #9715) were purchased from Cell Signaling Technology; α-tubulin (1:200; #sc12462-R) were purchased from Santa Cruz. Antibodies for IP and RIP: against MATR3 (#A300-591A, 2 μg) and MTR4 (#A300-614A, 3 μg) were purchased from Bethyl; GFP (#SAB4200681, 5 μg) were purchased from Sigma; FLAG (#A2220) were purchased from Millipore; rabbit IgG were purchased from GeneTex (#GTX35035, 3 μg) and mouse IgG were purchased from Millipore (#CS200621, 5 μg). Antibodies for immunofluorescence: against MATR3 (1:200, #MABN1587) and PSF (1:200; #P2860) were purchased from Sigma; MTR4 (1:200, #A300-614A) were purchased from Bethyl.
Validation	All antibodies were commercially sourced and were validated by their respective suppliers

Eukaryotic cell lines

Policy information about cell lines and Sex and Gender in Research

Cell line source(s)	The Jurkat (ARP-177) and J-Lat 9.2 (ARP-9848) cell lines were obtained from the AIDS Research and Reference Reagent Program (NIAID, NIH), now resourced by BEI Reagent Resources. Human embryonic kidney epithelial-like cells HEK 293T (ATCC #CRL-3216) and Lenti-X™ 293T (Takara Cat# 632180).
Authentication	J-Lat 9.2 cells were authenticated by the Verdin and Greene Laboratories at the Gladstone Institutes by PCR assays with species-specific primers. HEK293T cells were authenticated by the ATCC and Lenti-X 293T by karyotype analysis. None of the cell lines used were authenticated after buying from NIH (Bei Resources), ATCC or Takara.
Mycoplasma contamination	All cell lines were tested negative for mycoplasma.
Commonly misidentified lines (See ICLAC register)	No commonly misidentified cell lines were used in this study.

Clinical data

Policy information about clinical studies

All manuscripts should comply with the ICMJE guidelines for publication of clinical research and a completed CONSORT checklist must be included with all submissions.

Clinical trial registration	*Provide the trial registration number from ClinicalTrials.gov or an equivalent agency.*
Study protocol	*Note where the full trial protocol can be accessed OR if not available, explain why.*
Data collection	*Describe the settings and locales of data collection, noting the time periods of recruitment and data collection.*
Outcomes	*Describe how you pre-defined primary and secondary outcome measures and how you assessed these measures.*

Plants

Seed stocks	*Report on the source of all seed stocks or other plant material used. If applicable, state the seed stock centre and catalogue number. If plant specimens were collected from the field, describe the collection location, date and sampling procedures.*
Novel plant genotypes	*Describe the methods by which all novel plant genotypes were produced. This includes those generated by transgenic approaches, gene editing, chemical/radiation-based mutagenesis and hybridization. For transgenic lines, describe the transformation method, the number of independent lines analyzed and the generation upon which experiments were performed. For gene-edited lines, describe the editor used, the endogenous sequence targeted for editing, the targeting guide RNA sequence (if applicable) and how the editor was applied.*
Authentication	*Describe any authentication procedures for each seed stock used or novel genotype generated. Describe any experiments used to assess the effect of a mutation and, where applicable, how potential secondary effects (e.g. second site T-DNA insertions, mosiacism, off-target gene editing) were examined.*

Flow Cytometry

Plots

Confirm that:

- [x] The axis labels state the marker and fluorochrome used (e.g. CD4-FITC).
- [ ] The axis scales are clearly visible. Include numbers along axes only for bottom left plot of group (a 'group' is an analysis of identical markers).
- [ ] All plots are contour plots with outliers or pseudocolor plots.
- [x] A numerical value for number of cells or percentage (with statistics) is provided.

Methodology

Sample preparation	J-Lat 9.2 were collected 24 h after stimulation, centrifuged, washed in PBS, and resuspended in 3.7% paraformaldehyde (PFA; Sigma #P6148) in PBS for fixing. After 30 min, cells were washed twice in PBS.
Instrument	BD LSR Fortessa.
Software	BD FACSDiva 8.0
Cell population abundance	At least 10000 cells were counted per condition.
Gating strategy	Samples were aquired on BD LSR Fortessa analyzer and gates were set using the untimulated J-Lat 9.2 control sample. the gating strategy is FFS-A/SSC-A for cells then, FSC-H/FSC-A for single cells, FSC-W/GFP-A as shown in Suppl. Fig. 1

- [x] Tick this box to confirm that a figure exemplifying the gating strategy is provided in the Supplementary Information.
